# The Functions of BMP3 in Rabbit Articular Cartilage Repair

**DOI:** 10.3390/ijms161125937

**Published:** 2015-10-29

**Authors:** Zhe Zhang, Wenyu Yang, Yiting Cao, Yanping Shi, Chen Lei, Bo Du, Xuemin Li, Qiqing Zhang

**Affiliations:** 1 The Key Laboratory of Biomedical Material of Tianjin, Institute of Biomedical Engineering, Chinese Academy of Medical Sciences, Peking Union Medical College, Tianjin 300192, China; zhangzhe523575127@163.com (Z.Z.); Yang_eve@163.com (W.Y.); 15933766189@163.com (Y.C.); dubojlu@163.com (B.D.); 2 School of Chemistry and Chemical Engineering, Tianjin University of Technology, Tianjin 300384, China; yanpingshi@126.com (Y.S.); leichen788@163.com (C.L.)

**Keywords:** bone morphogenetic protein 3, articular cartilage, subchondral bone, repair

## Abstract

Bone morphogenetic proteins (BMPs) play important roles in skeletal development and repair. Previously, we found fibroblast growth factor 2 (FGF2) induced up-regulation of BMP2, 3, 4 in the process of rabbit articular cartilage repair, which resulted in satisfactory repair effects. As BMP2/4 show a clearly positive effect for cartilage repair, we investigated the functions of BMP3 in rabbit articular cartilage repair. In this paper, we find that BMP3 inhibits the repair of partial-thickness defect of articular cartilage in rabbit by inducing the degradation of extracellular matrix, interfering with the survival of chondrocytes surrounding the defect, and directly inhibiting the expression of *BMP2* and *BMP4*. Meanwhile BMP3 suppress the repair of full-thickness cartilage defect by destroying the subchondral bone through modulating the proliferation and differentiation of bone marrow stem cells (BMSCs), and directly increasing the expression of *BMP4*. Although BMP3 has different functions in the repair of partial and full-thickness defects of articular cartilage in rabbit, the regulation of BMP expression is involved in both of them. Together with our previous findings, we suggest the regulation of the BMP signaling pathway by BMP3 is essential in articular cartilage repair.

## 1. Introduction

Articular cartilage regeneration is still a major challenge in regenerative medicine for its sophisticated mechanism [1,2]. Many factors, such as cells, growth factors, and signaling pathway, are involved in the process of articular cartilage regeneration [3,4,5], and bone morphogenetic proteins (BMPs) and the BMP signaling pathway are important parts of them [6,7,8]. Previously, we found that fibroblast growth factor2 (FGF2) induced up-regulation of BMP2, 3, and 4 during rabbit articular cartilage regeneration, and resulted in a satisfactory repair effect [9]. Although BMP2/4 has shown a clearly positive effect for cartilage regeneration, this effect remains largely unknown for BMP3. Thus we investigated the functions of BMP3 in articular cartilage repair.

BMP3 accounts for almost 65% of total BMP in demineralized bone [10]. Unlike other BMPs, it has no osteogenic activity. It was first known as a negative regulator of bone density, which activated the TGF-β/activin pathway and antagonized the BMP pathway [11,12,13]. Then BMP3 was shown to regulate cartilage cell proliferation and endochondral bone formation through the type II receptor activin receptor type 2b (Acvr2b) during skeletal development [14,15]. Recently, it was reported that the expression of BMP3 was spatiotemporally controlled during development [16]. Although BMP3 is widely studied in skeletal development, its direct functions in the repair of articular cartilage defects are still unknown.

Articular cartilage defects can be divided into partial and full-thickness defects as well as osteochondral defects [17]. When defects involve only the articular surface without penetrating the tidemark, defined as partial-thickness defects, mesenchymal stem cells (MSCs) from the cartilage and synovial membrane are the main stem cells involved in its repair [18,19]. Defect which break the tidemark but do not involve the bone marrow are termed as full-thickness defect, which recruit bone marrow stem cells (BMSCs) to take part in the repair process [20,21]. BMP2 and BMP4 have been shown to be beneficial to the repair of these two kinds of defect through the BMP signaling pathway, which promote MSCs proliferation, chondrogenesis and stimulates extracellular matrix (ECM) synthesis [6,22,23]. As an important regulator of the BMP signaling pathway, we speculate that BMP3 may also regulate the articular cartilage repair just as it does in skeletal development.

In this paper, we report on our investigation of the functions of BMP3 in the articular cartilage repair of rabbit partial and full-thickness defects together with its functions via regulation of the BMP signaling pathway in chondrocytes and BMSCs *in vitro*.

## 2. Results

### 2.1. The Effect of BMP3 on Partial-Thickness Defects

Although the effect of BMP3 on bone development is clear [13,14,15], its effect on articular cartilage repair is still unknown. Thus, in this study, partial-thickness defects were created and implanted with BMP3-loaded collagen membrane, to study the functions of BMP3 on articular cartilage repair.

**Figure 1:**
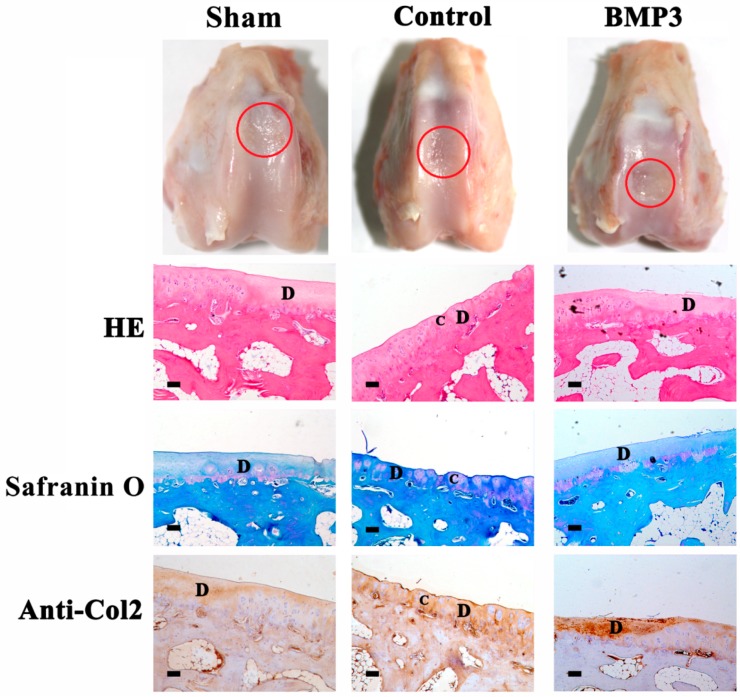
Macroscopic and histological examinations of the repaired tissue in partial-thickness defect at eight week post-operation. The red circle regions are the defects of articular cartilage. Sections were stained with hematoxylin and eosin (HE), Safranin O, or immunohistochemical collagen type II (Anti-Col2). Sham, sham-operated group; Control, group only treated with collagen membrane without BMP3 as negative control; BMP3, group treated with the collagen membrane and BMP3 at 100 ng/cm^2^, respectively. D and C stand for defect area and chondrocyte-like cells, respectively. Scale bar is 100 μm.

After eight weeks, the tissues were collected and observed macroscopically at first (Figure 1). There were no obvious differences among all groups. The defects were identifiable with whitish tissues and their margin can be easily identified from the native cartilage. Then the tissues were examined histologically (Figure 1). None of the three groups showed obvious tissue restoration, fibrocartilage formation or subchondral bone deformation. And there were no significant differences (*p* > 0.05) in the statistically scoring of Safranin O staining among the three groups (data not shown). In sham group, only few chondrocytes with abundant destroyed ECM can be seen under the defect, which was weak in Anti-Col2 immunohistochemistry staining and negative Safranin O staining. In the control group, although the repair effect of collagen membrane was limited, chondrocyte-like cells proliferated and aggregated in the injured matrix obviously, and the matrix showed much regular staining for both Anti-Col2 immunohistochemistry and Safranin O. The BMP3 treated group was similar to the sham group, in which cell proliferation and aggregation was inhibited. Fewer chondrocytes could be identified under the defect, and the ECM degradation was obvious. These results indicate that the spontaneous repair ability of articular cartilage in partial-thickness defect is poor and BMP3 inhibits the repair of partial-thickness defect.

### 2.2. The Effect of BMP3 on Full-Thickness Defect

**Figure 2:**
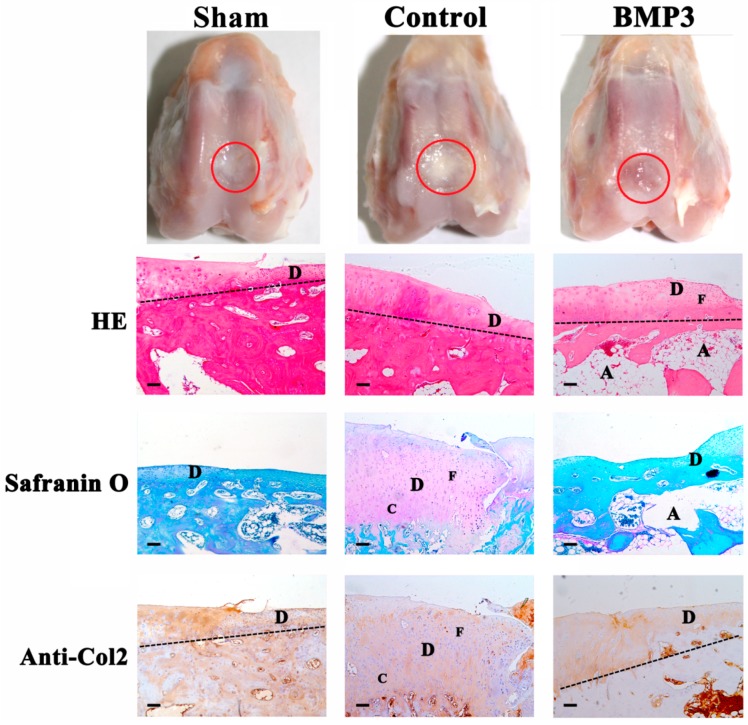
Macroscopic and histological observation of the repaired tissue in full-thickness defect at eight weeks post-operation. The red circle regions are the defects of articular cartilage. Sections were stained with HE, Safranin O, or immunohistochemical collagen type II (Anti-Col2). Sham, sham-operated group; Control, group only treated with collagen membrane without BMP3 as negative control; BMP3, group treated with the collagen membrane and BMP3 at 100 ng/cm^2^, respectively. The dash line stands for the boundary between articular cartilage and subchondral bone. D, C, F, A stand for defect area, chondrocyte-like cells, fibrocartilage cells, cavities, respectively. Scale bar is 100 μm.

We further investigated the effect of BMP3 on full-thickness articular cartilage defect through articular cartilage and subchondral bone, which integrate as a functional unit for joint movement. Macroscopically, the defects of both the sham and control group were filled with irregularly whitish fibrous tissues, while the BMP3 group was still partly filled and the margin was distinguishable from the normal cartilage (Figure 2). Histologically, fibrocartilage formation was obvious in the sham group; fibrocartilage cells as well as chondrocyte-like cells could be identified in the control group (Figure 2). For the BMP3 group, only fibrocartilage cells instead of chondrocyte-like cells appeared at the margin of defect. Worse still, even fibrocartilage formation was inhibited in BMP3 treated group that their defect was still partly filled. From Figure 2, it can be seen that the level of subchondral bone was significantly raised by BMP3. However, the structure of subchondral bone was destroyed, and large cavities can be seen. Together, these results indicated that BMP3 suppresses the repair of full-thickness defect by destroying the subchondral bone.

### 2.3. The Effect of BMP3 on Chondrocyte Proliferation and ECM Synthesis

**Figure 3:**
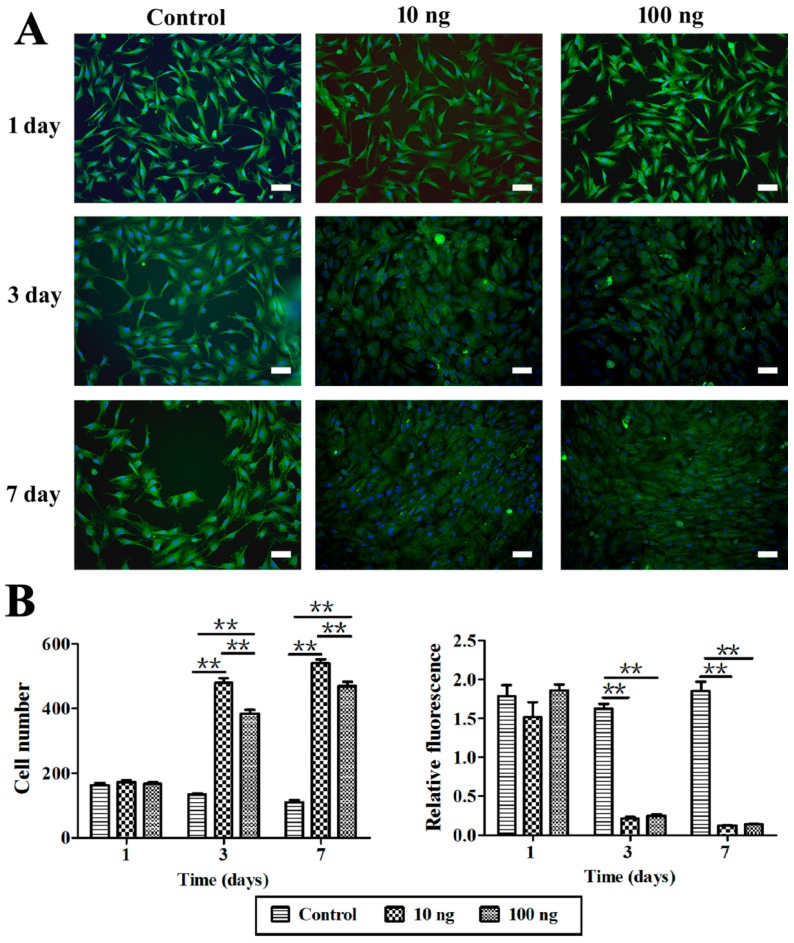
Observation and analysis of chondrocytes *in vitro* by high content screening. (**A**) The immunofluorescence image of chondrocytes, scale bar is 100 μm; (**B**) The cell number of chondrocytes content by nucleus and the calculated mean intensity of collagen type II according to the number of DAPI stained cells. 10 ng means 10 ng/mL, 100 ng means 100 ng/mL BMP3 in the culture media; control, without BMP3. Mean ± SD, *n* = 3; ** *p* < 0.01.

**Figure 4:**
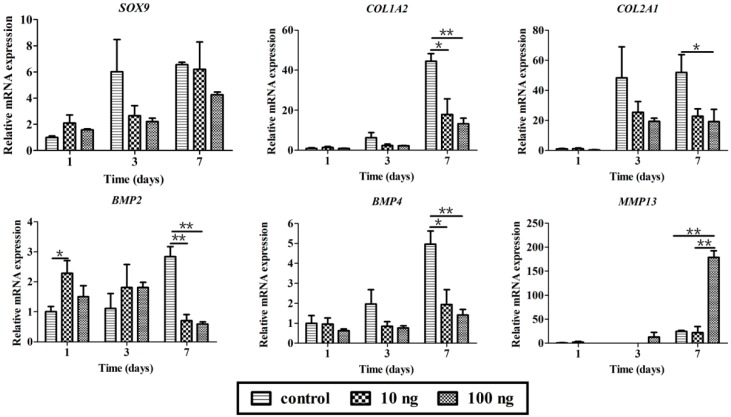
Effects of BMP3 on gene expression of chondrocytes *in vitro*. 10 ng means 10 ng/mL, 100 ng means 100 ng/mL BMP3 in the culture media; control, without BMP3. Mean ± SD, *n* = 3; * *p* < 0.05, ** *p* < 0.01.

To further demonstrate the bioactivity of BMP3, rabbit chondrocytes were isolated and treated with BMP3 *in vitro*. First, the cell morphology and number as well as type II collagen content were investigated by high content screening. From Figure 3, it could be seen that BMP3 can obviously increase the cell number compared with the control group. According to the number of DAPI stained cells, the calculated mean intensity of type II collagen was decreased significantly in BMP3-treated groups at day three and seven. Then the effects of BMP3 on gene expression were investigated as shown in Figure 4. The expression of *COL1A2* and *COL2A1*, but not *SOX9*, decreased significantly at day seven when treated with BMP3. In addition, the expression of *MMP13* at day seven was dramatically increased. These results indicate that BMP3 may induce chondrocyte proliferation, but also inhibit the synthesis, and accelerate the degradation, of ECM. Additionally, it is worth noting that BMP3 significantly decreased the expression of *BMP2* and *BMP4* at day seven, which indicates that BMP3 not only antagonizes the canonical BMP and activin pathways, but also directly suppresses the expression of *BMP2* and *BMP4*.

### 2.4. The Effect of BMP3 on BMSCs Proliferation and Differentiation

**Figure 5:**
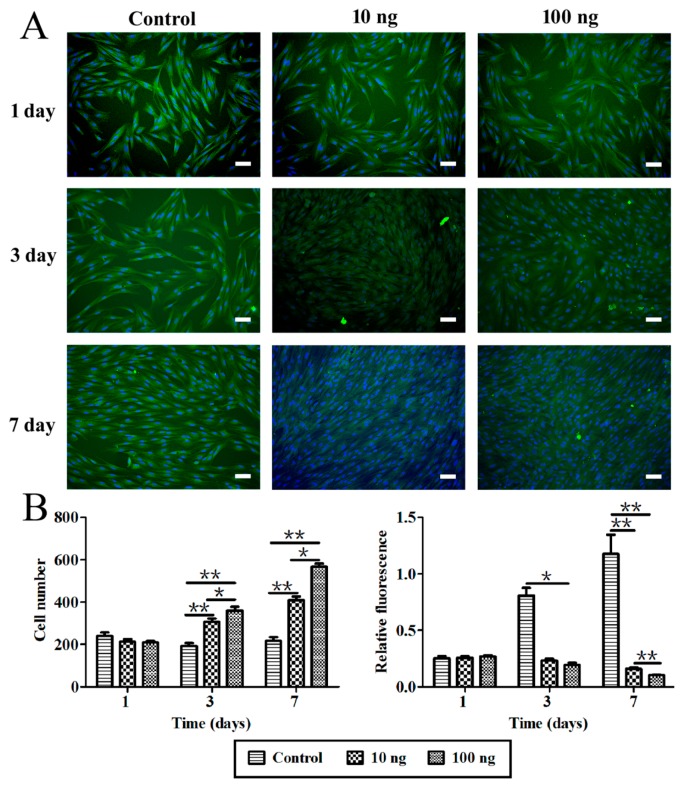
Observation and analysis of BMSCs *in vitro* by high content screening. (**A**) The immunofluorescence image of BMSCs, scale bar is 100 μm; (**B**) The cell number of BMSCs content by nucleus and the calculated mean intensity of collagen type II according to the number of DAPI-stained cells. 10 ng means 10 ng/mL; 100 ng means 100 ng/mL BMP3 in the culture media; control, without BMP3. Mean ± SD, *n* = 3; * *p* < 0.05, ** *p* < 0.01.

**Figure 6:**
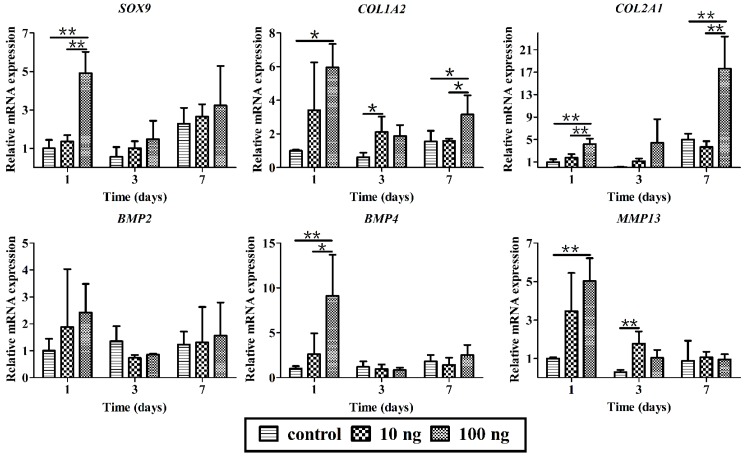
Effect of BMP3 on gene expression of BMSCs *in vitro*. 10 ng means 10 ng/mL; 100 ng means 100 ng/mL BMP3 in the culture media; control, without BMP3. Mean ± SD, *n* = 3; * *p* < 0.05, ** *p* < 0.01.

BMSCs play important roles in articular cartilage repair. Therefore, we investigated the effect of BMP3 on BMSCs. From Figure 5, it can be seen that cell numbers of BMP3-treated groups were more than the control groups at day three and seven, which indicates that BMP3 promotes the proliferation of MSCs, as previous reported [24,25,26]. However, according to the number of DAPI-stained cells, the calculated mean intensity of type II collagen was obviously decreased by BMP3 at day three and seven. To explain this phenomenon, their relative gene expression was investigated. BMP3 showed potential to induce BMSCs differentiated into chondrocyte-like cells with the up-regulated expression of *COL2A1*, *COL1A2* and *SOX9* (Figure 6). In particular, for 100 ng BMP3 group, *COL2A1* expression was continuously up-regulated. These results indicated that BMP3 can induce chondrogenic differentiation of BMSCs to a certain degree. But BMP3 also up-regulated *MMP13* significantly, which could be responsible for the decreased intensity of type II collagen (Figure 5). In addition, BMP3 only significantly regulated the expression of *BMP4* just one day after treatment, but had no influence on *BMP2*. Taken together, BMP3 may promote proliferation of BMSCs and chondrogenic differentiation, but the increased expression of *MMP13* may not be beneficial for chondrogenesis.

## 3. Discussion

In the present study, we investigated the effect of BMP3 on articular cartilage repair. BMP3 inhibits the repair of partial-thickness cartilage defects by inducing the degradation of the extracellular matrix and interfering with the survival of chondrocytes surrounding the defect. Meanwhile BMP3 also suppresses the repair of full-thickness cartilage defects by destroying the subchondral bone. Further studies revealed that BMP3 increased the proliferation of chondrocytes and BMSCs. However, it inhibits the synthesis or fastens the degradation of type II collagen in them. The results of gene expression assays show that BMP3 impairs survivial of chondrocytes and inhibits the differentiation of BMSCs.

BMP3 plays important roles in bone formation and is modulated by mechanical loading [27,28,29]. BMP3 is strongly expressed in the developing perichondrium and improved cartilage cell proliferation by modulating BMP signaling through the type II receptor activin receptor type 2b (Acvr2b), which further affected bone formation [15]. Moreover, the expression of BMP3 in the special layer of articular cartilage is correlated with mechanical loading [30]. In addition, BMP3 may also play important roles in articular cartilage repair. Previously, we found exogenous FGF2 induced the up-regulation of endogenous BMP2, 3, 4 at a very early stage and promoted the articular cartilage repair in rabbit [9], which means BMP3 together with BMP2/4 play critical roles in articular cartilage repair. However, to our knowledge, there is no report on the role of BMP3 in articular cartilage repair.

Here we found that exogenous BMP3 inhibits partial-thickness articular cartilage repair through regulating chondrocytes, which is associated with the BMP signaling pathway by regulating the expression of *BMP2* and *BMP4*. BMP3 impaired the poor spontaneous repair ability of articular cartilage by inducing the degeneration of ECM and inhibiting the survival of chondrocytes. As BMP3 down-regulated the expression of *COL2A1* and *COL1A2* and up-regulated the expression of *MMP13* for chondrocytes *in vitro*, it will lead to the matrix degradation of cultured chondrocytes and cartilage. Although it increases the proliferation of chondrocytes *in vitro*, the destroyed ECM can inhibit the survival of chondrocytes [31,32]. In addition, BMP3 down-regulated the expression of both BMP2 and BMP4 in cultured chondrocytes, which indicates that BMP3 may alter the BMP signaling pathway indirectly.

We also found that BMP3 suppresses the repair of full-thickness cartilage defect by destroying the subchondral bone via modulating the expression of BMPs. Since BMP3 was reported to regulate chondrocytes and MSCs proliferation by modulating of TGF-beta/activin signaling through Acvr2b [14,24], the proliferation of BMSCs induced by BMP3 may also be due to its modulation through the TGF-beta/activin signaling pathway. Moreover, BMP3 increased the expression of *SOX9*, *COL1A2*, *COL2A1* and *MMP13* in BMSCs. Given that BMP3 inhibits the synthesis of collagen type II, and the recent report that it promotes the differentiation of MSCs into a nucleus pulposus-like phenotype [26], we can draw the conclusion that BMP3 has limited effect on chondrogenesis in BMSCs. The significant effect on proliferation may lead to the increasing level of subchondral bone. And BMP3 may inhibit fibrocartilage formation so that the defect of BMP3 treated group was still only partly filled. In addition, it was reported thatBMP3 suppresses osteoblast differentiation of BMSCs and that BMP3 is regarded as a negative regulator of bone density [12,33]. In full-thickness cartilage defects, BMP3 may inhibit osteoblast differentiation of BMSCs and suppress bone remodeling, as seen in the large cavities shown in the subchondral bone. Interestingly, BMP3 may also regulate the BMP signaling pathway through increasing the expression of *BMP4*.

**Figure 7:**
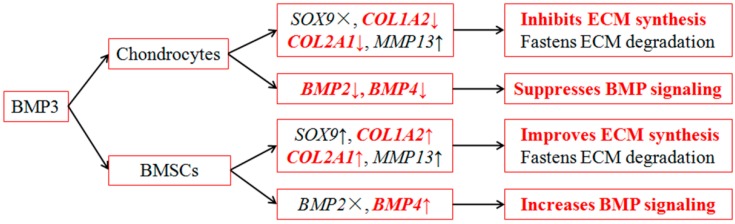
The functions of BMP3 between chondrocyte and BMSCs. ×, ↑, ↓ stand for no effect, up-regulate, down-regulate, respectively.

Additionally, six genes are chosen to investigate the effect of BMP3 between chondrocyte and BMSCs. *SOX9* plays a critical role in early chondrocyte differentiation [34]. *COL1A2*, *COL2A1*, and *MMP13* are correlated with the synthesis and degradation of collagen type I and II [35], which affect extracellular matrix homeostasis and cartilage repair [36]. *BMP2* and *BMP4* are crucial components of the BMP signaling pathway [37], and were chosen to investigate the indirect effects of BMP3 on BMP signaling. Figure 7 shows the functions of BMP3 in chondrocytes and BMSCs. For chondrocytes, BMP3 down-regulates the expression of *COL1A2* and *COL2A1* to inhibit the synthesis of ECM, meanwhile it also down-regulates the expression of *BMP2* and *BMP4* to suppress BMP signaling. For BMSCs, BMP3 up-regulates the expression of *COL1A2* and *COL2A1* to improve the synthesis of ECM, meanwhile it also up-regulates the expression of *BMP4* to increase BMP signaling.

## 4. Experimental Section

### 4.1. Preparation of BMP3-Loaded Collagen and Collagen (Col)/Hydroxyapatite (Hap) Membranes

BMP3-loaded collagen and Col/Hap membranes were produced from collagen and Col/Hap suspension using a freeze-drying technique.BMP3 was purchased from Sigma-Aldrich (catalog number: SRP4573, St. Louis, MO, USA), native insoluble type I collagen was isolated from bovine Achilles tendon in our laboratory [38]. Hydroxyapatite was added to 1% (*w*/*v*) collagen solution by the final weight ratio of 80/20 (hydroxyapatite to collagen). The homogenized suspension was put into a polytetrafluoroethylene container and placed in a freezer at −80 °C. The mixtures were then lyophilized at −55 °C and 5 Pa for 2 days (ALPHA 1–2 LD plus, Christ, Osterode, Germany). The membranes were sterilized by gamma irradiation (25 kGy). The solution of BMP3 in phosphate buffered saline (PBS, pH 7.4) was dropped slowly into the collagen and Col/Hap membranes and then air-dried at room temperature on a clean bench. The diameter of the membranes was approximately 1.5 cm, meanwhile the thickness were approximately 1 and 2 mm for collagen and Col/Hap membranes, respectively. The amount of BMP3 was 100 ng/cm^2^ of the membrane.

### 4.2. Animal Model

Animal experiments were in compliance with the guidelines made by the Animal Committee of Tianjin, China. The rabbits were allowed to accommodate the experimental environment for 1 week before surgery. Skeletally mature Japanese white rabbits (*n* = 12, 2.7–3.5 kg) were anesthetized by intramuscular administration of Su-Mian-Xin II (HuaMu Animal Health Care Products Co., Ltd., Jilin, China) at the dose of 0.2 mg/kg. Half doses of Su-Mian-Xin II were given to maintain the anesthesia at half hour intervals during operation. The patella was dislocated laterally after skin incision.

To evaluate the effect of BMP3 on repair, partial and full thickness defects were created by scalpel and trephine in left and right articular cartilage, respectively. For partial-thickness defect, the cartilage surface was shaved to make a mid-deep layer defect. For full-thickness defect, we created a cylindrical defect of 2 mm thickness in the center of the trochlear groove. For each defect, the rabbits were divided into three groups and transplanted with (1) membranes with BMP3 and (2) membranes without BMP3 as the control group and (3) sham group without membranes. At last, the joint capsule, fascial layer, and skin were sutured, respectively. The rabbits were euthanized at eight weeks post-injury by injecting anesthetic agent, and four knees from each group were dissected.

### 4.3. Macroscopic and Histological Evaluation

The repaired cartilage samples were examined macroscopically at first. Then they were fixed in 4% paraformaldehyde solution. The fixed specimens were decalcified by ethylenediaminetetraacetic acid (EDTA) solution for 1 week, and embedded in paraffin wax. Sagittal sections (4-μm thick) were cut through the centre of the defect. Finally, the specimens were stained with hematoxylin and eosin (HE) and safranin O/fast green (Safranin O) for histological evaluation, and examined by a light microscope (DMRB, Leica, Germany).

### 4.4. Immunohistochemistry for Collagen Type II

The specimens were deparaffinizated and dehydrated by xylene and graded alcohols, respectively. Then citric acid buffer and 3% H_2_O_2_ were added for antigen retrieval, followed by treatment with PBS (pH 7.4) containing 10% goat serum to block nonspecific staining as reported [9]. After incubation with mouse monoclonal anti-type II collagen primary antibodies (Santa Cruz Biotechnology, Santa Cruz, CA, USA) at 4 °C overnight, the specimens were incubated with a secondary goat anti-mouse IgG antibody (EarthOx, Millbrae, CA, USA) at 37 °C for half an hour. Finally, they were immunostained with peroxidase-conjugated streptavidin and (3, 3′-diaminobenzidine) Substrate Kit, and counterstaining with hematoxylin.

### 4.5. Isolation of Articular Chondrocytes and BMSCs

To further elucidate the mechanism of BMP3 on articular cartilage repair, chondrocytes and BMSCs were isolated and cultured *in vitro*. Five 4-week-old Japanese white rabbits were euthanized with an overdose of anesthetic agent. Articular cartilage was removed under aseptic conditions and washed with PBS. Chondrocytes were extracted by digesting the cartilage chips with type II collagenase (Sigma, St. Louis, MO, USA) for 14 h at 37 °C and 5% CO_2_ as reported [39]. The cell suspension was filtered through a 70-μm nylon mesh, and the isolated chondrocytes were washed with PBS. BMSCs were extruded with culture medium (high-glucose DMEM supplemented with 10% fetal bovine serum and 1% penicillin/streptomycin) and isolated by short-term adherence to plastic as described previously [40]. Culture medium was changed twice a week, and cells at passage 3 were used.

### 4.6. Immunofluorescence Staining of Collagen Type II

Chondrocytes and BMSCs were plated in 96-well plates (catalog number: 3603; Corning, New York, NY, USA) at 1 × 10^5^ cells/cm^2^ and cultured with BMP3 at concentrations of 0, 10 and 100 ng/mL. Culture medium without BMP3 was defined as control group. Chondrocytes and BMSCs cultured for 1, 3 and 7 day were fixed in 4% paraformaldehyde solution overnight at 4 °C. Then the cells were washed with PBS containing 0.1% Tween 20 (PBST) (3 times at 30 min each). To block the non-specific binding sites, Bovine Serum Albumin was used for 1 min and washed with PBST (3 times). Mouse anti-COLII primary antibody (Abcam Inc., Cambridge, MA, USA ab3092) was added and incubated at 4 °C overnight. Cells were washed with PBST (3 times), and incubated in anti-mouse IgG-FITC secondary antibody (Abcam Inc., Cambridge, MA, USA ab6785). After that, cells were washed with PBST (3 times). They were incubated in 50 mg/mL DAPI for 5 min at room temperature and washed again as reported [41]. The fluorescence-stained cells were visualized and analysis by high content screening (GE Healthcare, Buckingham, UK).

### 4.7. RNA Extraction

Chondrocytes and BMSCs were plated in 6-well plates at 1 × 10^5^ cells/cm^2^ and cultured with BMP3 at concentrations of 0, 10 and 100 ng/mL. Culture medium without BMP3 was defined as control group. Culture medium was changed twice a week. On days 1, 3 and 7, total RNA was extracted using an E.Z.N.A. HP Total RNA Kit (Omega Bio-Tech, Victoria, BC, Canada) following the manufacturer’s instructions. The quantity and quality of RNA samples were assessed by Nanovue (GE Healthcare, London, UK), and only samples with a ratio of A_260_:A_280_ higher than 1.8 were used in subsequent experiments.

### 4.8. Reverse Transcription Quantitative Real-Time Polymerase Chain Reaction (RT-qPCR)

RT-qPCR was used to determine the relative mRNA levels of genes involved in articular cartilage repair. First-strand complementary DNA (cDNA) was synthesized using a GoScript™ Reverse Transcription reagent kit (Promega, Southampton, UK) as described by the manufacturer. The samples were stored at −20 °C prior to RT-qPCR.

The PCR amplification on a subset of the cDNA samples using glyceraldehyde-3-phosphate dehydrogenase (GAPDH) as reference genes to confirmed successful reverse transcription. The sequences of the primers are listed in Table 1. RT-qPCR was performed using a Hot Start Fluorescent PCR Core Reagent Kit (Promega, Southampton, UK) according to the manufacturer’s instructions on an ABI PRISM 7500 Real-Time PCR System (Applied Bio-systems, Foster, CA, USA). Each reaction was performed in a 20-μL total volume with 7 μL PCR-H_2_O, 0.5 μL forward primer (0.2 μM), 0.5 μL reverse primer (0.2 μM), and 10 μL Hot Start Fluo-PCR mix, to which 2 μL of cDNA was added as the PCR template.

For all the genes, the PCR conditions were as follows: pre-denatured at 95 °C for 2 min, and then denatured at 95 °C for 15 s and annealed/extended at 60 °C for 60 s for 40 cycles. PCR products were quantified by the cycle threshold (*C*_t_) value. Each gene was normalized to their relative *GAPDH* levels using the 2^−ΔΔ*C*t^ method. The mRNA levels were normalized to the control group.

**Table 1:** A list of RT-qPCR primers and product sizes. (F = Forward, R = Reverse).

Gene	Reference Sequence	Primer Sequence (5′ to 3′)	Product Size (bp)
*GAPDH*	NM001082253	F: GCC CCT CTT CAC AGT TTC CA	97
		R: GCT GTC GAG ACT TTA TTG ATG GT	
*SOX9*	XM008271763.1	F: CAA GAA AGA CCA CCC GGA CT	123
		R: GCC TTG AAG ATG GCG TTG GG	
*COL1A2*	NM001195668.1	F: CCA TCT CGT TTG CCC TTC CT	80
		R: GGG CCA ACG TCC ACA TAG AA	
*COL2A1*	NM001195671	F: TGC AGG AGG GGA AGA GGT AT	123
		R: GGC AGT CCT TGG TGT CTT CA	
*BMP2*	NM001082650	F: CGC CTC AAA TCC AGC TGT AAG	80
		R: GGG CCA CAA TCC AGT CGT T	
*BMP4*	NM001195723	F: ACC GAA TGC TGA TGG TCG TT	84
		R: TCT TCC CCG TCT CAG GTA TCA	
*MMP13*	NM001082037	F: CTG ACT AGG AAG CGG AAG CC	125
		R: ACA CCT GGC TGC ATC TTG AA	

### 4.9. Statistical Analysis

All data were expressed as the mean ± standard deviation (SD). One-way analysis of variance (ANOVA) was performed to assess the statistical significance of differences between groups. The general linear model was tested for homogeneity of variance (Levene’s test). Least-significant difference (LSD) and Student-Newmnan-Keuls (SNK) tests were used to assess the statistical significance among all treatments. The results of statistical analyses were performed using SPSS (version 16, SPSS Inc., Chicago, IL, USA) software based on statistically independent observations, and differences were considered statistically significant when the *p*-value was less than 0.05.

## 5. Conclusions

BMP3 inhibits the repair of partial-thickness cartilage defects by inducing degradation of the extracellular matrix and interfering with the survival of chondrocytes surrounding the defect. Meanwhile BMP3 suppresses the repair of full-thickness cartilage defects by destroying the subchondral bone. Given our previous findings, BMP2, 3, and 4 upregulation by exogenous FGF2 resulted in outstanding repair effect. We speculate that the successful repair of articular cartilage may require regulation of the BMP signaling pathway by BMP3.
